# Miscellanies

**Published:** 1834-10-01

**Authors:** 


### MISCELLANIES.

### The North-West London Self-supporting Dispensary.

We are not exactly of the Chancellor's school, nor do we think it absolutely indispensable that a man should break his ribs, his head, or his skull, in order to be qualified to receive gratuitous medical assistance. No doubt, the knowledge that a pauper fund exists is injurious, in some measure, medically as politically. But the balance of good is vastly in its favour; and medical charity, on an extensive scale, is probably attended with a smaller amount of ultimate mischief, than any other form of this offspring of humanity and kindliness. There is one consideration in favour of these charities, which appears to have been totally lost sight of—their importance as a means of medical education. Were the Chancellor seriously to attempt to put down all medical charities, save and excepting hospitals for accidents, we should like to know how, where, and when the professional youth should receive their education? The Malthusians, we ween, would look exceeding blue, did they find that themselves and their families were deprived of decent professional assistance—that their wives must be consigned to the science of midwives—and their own inflammations, and choleras, and fevers, be entrusted to the hands of a set of empirics. Then the titled railers at a charity fund would import their physicians from some foreign land, where ignorance of the true principles of political economy perpetuated hospitals, and maintained institutions for the halt and blind. Yet we need not throw away words on the affair, for the whole is an idle, shapeless dream, incapable of form, and impossible of occurrence.*

Whilst we laugh at the idea of abolishing hospitals, we feel that it is politic and in every way desirable, to prevent the lower classes from leaning too much on the certainty of gratuitous advice. The penny earnings squandered in the destruction of life by gin, might be usefully and honourably appropriated to securing the means of restoring health, or alleviating suffering, at a future day. That which philanthropy might wish, active benevolence, it would seem, has effected. In many country districts, self-supporting dispensaries have been founded, and are flourishing. The labourer and the mechanic have been taught to believe, and have discovered by experience, that a very few pence, judiciously applied, will preserve their independence, maintain their health, and draw the bold line between honourable self-respect, and the cringing, spurned, and wretched degradation of the pauper. Oh! it was a noble wish, to plant and to foster the seed of forethought in the bosom of the poor man, and well may it succeed.

The North-west London Self-supporting Dispensary is the offspring of those which have preceded it in the provinces. It is patronized by the noble and the wealthy, the Bishop of London being its president, and a long array of lords and gentlemen lending it their countenance and name. Its medical officers are of high respectability, and nothing wearing even the faintest colour of a job sticks any where about it.* The experiment of establishing a self-supporting dispensary in London is fairly made, and will be fully tried. We will glance at some of the

---

* Some of our contemporaries, not content with the medical portion of the question, have taken into consideration the Poor-law Bill. The passions and the politics of the newspaper have invaded the pages which should be exclusively devoted to science. The thing may be harmless—it is certainly absurd.

* Amongst the medical officers are Dr. Copland—Dr. C. B. Williams, the author of some excellent observations upon auscultation—Mr. Copland Hutchison—and Mr. Cæsar Hawkins.

more prominent features of the institution.

The following outline of the plan is presented in a circular, printed by the managing committee.

The Institution is supported by two distinct funds—the *Honorary* and the *Ordinary* Fund.

The Honorary Fund is derived from the subscriptions and donations of the benevolent, and is applied to defray the expenses of the Establishment, and the dispenser's salary.

The Ordinary Fund consists of the small periodical payments of the poor subscribers (one penny a week for each adult, and one halfpenny for each child, or one penny for all the children of a family, where there are more than two), and is devoted to the purchase of drugs, and the remuneration of the ordinary medical attendants.

Before persons can be admitted to the benefits of the Institution as ordinary subscribers, their circumstances are investigated by the Secretary and a Sub-committee, to ascertain that they really belong to that class for which the Institution is formed. The proceedings of the Sub-committee, as well as the general business of the Institution, are under the direction of the Committee. The power of controlling the Committee, of altering or making laws, and of electing or removing officers, is vested in the Governors (donors of ten guineas, or Annual Subscribers of one guinea), who meet once a year, or oftener, as circumstances may require.

Four medical officers in ordinary, who are general practitioners, attend in turn daily at the Dispensary, and visit, at their own homes, those of the ordinary subscribers who are too ill to come out. There are, besides, five consulting medical officers, physicians and surgeons, who give their services gratuitously, in cases of difficulty or danger.

This plan is adopted, with some modifications, from that of the Coventry Self-supporting Dispensary; and, as a proof of the practical success of that Establishment, it may be stated, that during the last year, which was its third, 1668 patients had been attended, and the free members' or ordinary fund amounted to £400. 12s. of which £112. 12s. were paid for drugs, and the remainder divided among the medical attendants.

We shall select such of the rules affecting ordinary subscribers, the class to be relieved, as develope the more important parts of the constitution of this self-supporting dispensary.

"I. The Ordinary Subscribers shall consist of small tradesmen, working persons, individuals of small income, and servants, their wives and children, not receiving parish relief, and who are unable to pay for medical advice in the usual manner, residing within the following limits:—The New Road, Oxford Street, the Edgeware Road, Portland Place, and Regent Street.

II. Any such person wishing to become an Ordinary Subscriber, must apply to the Secretary, who shall enter the name, age, residence, and occupation; the application will then be taken into consideration by the Sub-committee, and if found eligible, the applicant will be admitted, and receive a ticket on paying one month's subscription.

III. Every Ordinary Subscriber above fourteen years of age shall pay one penny, and under that age one halfpenny a week, except in a family with more than two children, when one penny a week shall be considered sufficient for all under fourteen years of age. Female servants shall pay five shillings a year, and male servants seven shillings, in not less than half-yearly payments.

IV. The payments of the Ordinary Subscribers shall be made at the Dispensary in advance, weekly or for any longer period. No one will be entitled to the benefits of the Institution if in arrear: and each family shall pay a fine of one penny for the arrear of every week. If any Ordinary Subscriber shall be more than four weeks in arrear, his or her name shall be erased from the books.

V. No one actually labouring under sickness can be admitted an Ordinary Subscriber, unless two healthy persons above fourteen years of age enter at the same time and each pay the whole year's subscription in advance. Any such person unable to procure two others to enter with him, shall, by paying ten shillings, be entitled to the privileges of an Ordinary Subscriber for three months; and may afterwards continue to be entitled to the benefits of the Institution by paying the usual rate of subscription."

Each patient may choose his or her medical attendant in the first instance; but he must not subsequently revoke his choice.

Any married Ordinary Subscriber being pregnant, may have the attendance of whichever Medical Officer in ordinary she may prefer, on depositing at the Dispensary half a guinea, either at once or by instalments, one month before her expected confinement.

Those who are sufficiently well, attend at stated hours at the dispensary—while those whose illness is too severe to permit such attendance, are visited at their own homes by the medical officers.

There is only one danger to be apprehended in the practical operation of these establishments. We all know the cruel indifference and neglect displayed by the subalterns, at hospitals and dispensaries, to the unhappy patients. Compelled to waste hour after hour in waiting to be seen, or in expecting their medicines, the industrious poor are heartlessly robbed of their time, their best possession, while their spirits are broken, and their maladies are aggravated, by exposure in cold and comfortless lobbies. Expostulation brings insult, and insult begets despair, till, too frequently, the patient is urged to discontinue the painful and perhaps injurious attendance—to turn with disgust from the cruel dole of insolent charity—and to submit, without a further struggle, to his fate. This is no exaggerated picture of the miserable hanger-on at a dispensary; and often have we heard a sickly mother bitterly complain, that her child and herself must perish unassisted, rather than suffer the intolerable delays and the hard-hearted arrogance of dispensary Dogberries. We fear that the small sums subscribed by the patients, in these new institutions, may tempt their menials to treat them as paupers are too often treated. The committee should keep a watchful eye on this, for slight as seems the evil, it cuts keen.

---

### Last Curriculum of the College of Surgeons.

We really cannot say whether this curriculum, promulgated in July of the present year, is merely a reiteration of former rules and regulations, or a genuine addition to the family of the college. Curricula have lately been so plenty, that we feel as much difficulty in distinguishing their features, names, and ages, as if we were placed amongst a series of twins. Whether the present be new or old, it is undoubtedly the last, and will constitute the law, we presume, for a season. There is tacked to it a recommendation which may merit a remark.

*Regulations of the Council respecting the Professional Education of Candidates for the Diploma.*

I. Candidates will be required to bring proof:—

1. Of being twenty-two years of age.

2. Of having been engaged five years in the acquirement of professional knowledge.

3. Of having studied Anatomy and Physiology, by attendance on Lectures and Demonstrations, and by Dissections, during two anatomical seasons.

4. Of having attended at least two courses of Lectures on Surgery, delivered in two distinct periods or seasons, each course to comprise not less than sixty Lectures.

5. Of having attended lectures on the Practice of Physic, on Chemistry and on Midwifery, during six months; comprising not less than sixty Lectures respectively, and on Botany and Materia Medica during three months.

6. Of having attended, during twelve months, the surgical practice of a recognised hospital in London, Dublin, Edinburgh, Glasgow, or Aberdeen; or for six months in any one of such hospitals, and twelve months in any recognised provincial hospital.

II. Members and Licentiates in Surgery of any legally constituted College of Surgeons in the United Kingdom, and Graduates in Surgery of any University requiring residence to obtain Degrees, will be admitted for examination on producing their Diploma, License, or Degree, together with proofs of being twenty-two years of age, and of having been occupied five years in the acquirement of professional knowledge.

N.B.—Certificates will not be recognised from any hospital unless the surgeons thereto, or a majority of them, be members of one of the legally constituted Colleges of Surgeons in the United Kingdom; nor from any school of Anatomy, Physiology, Surgery, or Midwifery, unless the respective teachers be members of some legally constituted College of Physicians or Surgeons in the United Kingdom.

Certificates will not be received on more than two branches of science from one and the same Lecturer, but Anatomy and Physiology, Demonstrations and Dissections, Materia Medica and Botany, will be respectively considered as one branch of Science.

In the Certificates of attendance on Hospital Practice, and on Lectures, the dates of commencement and termination are to be inserted in words at full length.

All the required Certificates are to be delivered at the College ten days before the Candidate can be admitted to Examination.

(By Order of the Council,)
Edmund Belfour, *Sec.*
July 10, 1834.

Few observations are necessary at present upon these regulations. The medical constitution is at this moment in so feverish and precarious a condition, that the Colleges might pause in the issue of their bulletins or prescriptions, and certainly it is unnecessary to contemplate with a very critical eye, directions and orders which may possibly be shortly over-ruled by the legislature. When we look at the regulations before us, we are struck with their inadequacy to ensure a high amount of surgical attainments. The young man of twenty-two, who has spent only five years in learning his profession, who has dissected for two seasons, walked a hospital for one year, and acquired such a smattering of surgery, physic, chemistry, midwifery, and botany, as the courses of lectures prescribed can confer—may possibly be adapted for a college examination, may probably be possessed of respectable acquirements, but assuredly is fit for little but the former, and cannot pretend to more than the latter. Yet we think that the circumstances to which we have alluded will excuse our pursuing the subject further.

Appended to the regulations is a circular addressed to lecturers, which conveys a sort of censure on them and on their pupils.

*Circular to Lecturers.*

Sir,—In consequence of the occasional irregularities that have taken place in the certificates, transmitted to the Court of Examiners of this College, I am directed by the Council to acquaint you; that it is their anxious wish to have some plan devised and adopted by the lecturers at the various medical schools of the United Kingdom, whereby the regular attendance of the students on the lectures at such schools may be enforced and registered so as to entitle the students to receive, and to justify the lecturers in giving, certificates of attendance, the accuracy of which may be relied upon by this College, and which the greatest vigilance and circumspection on the part of the lecturers cannot at all times secure under the present system, where there is a want of some such check and registry.

The Council will consider themselves obliged by your attention to their wishes, and will thankfully receive any suggestions you may be pleased to offer on the means which, in the opinion of yourself and your colleagues, may be best adapted to effect this important object.

I have the honor to be, Sir,
Your most obedient,
Humble servant,
Edmund Belfour, *Sec.*
July 2, 1834.

It is undoubtedly desirable that certificates of attendance upon lectures should have something like truth to recommend them. It is monstrous that a pupil should have it in his power to absent himself from lectures, and still be entitled to certificates of attendance on them. Yet surely the facility and the frequency of this practice form a biting commentary on the College examinations, or the "recognized" lectures. For our own parts, we almost think that the College might have spared itself the trouble of the circular. Were its own examinations searching, it would signify little what lectures the student had attended or had slurred. Under the expectation of a strict ordeal he must and he would repair to the sources of real information, the dissecting room, the hospital, or the lecture. And if the ordeal is not severe, the Council of the College may be well assured that their ingenuity will be frustrated by the stronger motives of pleasure, of indolence, or of disgust. Where the lecturer is calculated to convey instruction, his benches are comparatively seldom empty.

---

### Provincial Medical Schools.

James Collins, M.D. of Liverpool, has written a long letter in the Medical Gazette, avowedly for the purpose of exposing Provincial Schools of Medicine, and evidently with the view of satirizing a large class of his provincial brethren. We shall not attempt to analyze the motives of this letter-writing gentleman—we will not pronounce upon the taste, the candour, the professional conduct, or the gentlemanly feeling which characterize the remarkable production. The fearless *delator* of the wrong doings of his neighbours is doubtless immaculate himself—is unquestionably free from those sentiments of envy, hatred, and uncharitableness, which too often embitter the observation of success in which we are prevented from participating—is certainly actuated by zeal for the profession, and an ardent desire for the interests of science.

Yet the following picture might seem to display the exaggeration of the satirist, with some of his ill nature. It is in colours such as these, that Dr. Collins depicts the rise and the progress of the surgeons and physicians of Liverpool.

"The practice usually is, for those beginning to settle, to remain for some time unattached to any particular sect or party, and to observe in silence how the wind may blow, and how others got on before them. In the interim they are not idle; they are active in extending their acquaintances, and by pliancy of character fitting themselves for whatever may turn up, or for any vacancy in this or that congregation. Whatever people may say of the usual want of religion in medical men, here they are remarkably edifying. Some of them preach, pray, and assist as regular stewards of the vineyard, by word and example. Some read the service of their church in the streets on Sunday, as they pass along in their carriages or gigs, intimating thereby that they have not time for attendance at their church or chapel; others are very conspicuous leaders of class meetings; and many of them pray with their patients as regularly as they visit them, and hold prayer meetings at their own houses for them and others. In fact, few towns present such an instructive and edifying example as Liverpool. Here all sects have their favourite physician and surgeon; church and chapel differ little in this respect. Here every thing connected with religion or morals, bible and missionary meetings, temperance and providence societies, tract and religious associations, readily meet the support and countenance of medical men; and as the labourer is worthy of his hire, they have their reward in increased practice and connexion, or occasionally in a happy and lucrative matrimonial hit. It is no uncommon thing to see medical men here pass through all sects before they get one that answers. Some have begun their career as unitarians, then turned presbyterians, independents, methodists—in fact every thing before they finally settled down into their present character of high church folk; and I am satisfied would as easily revert again to the point whence they started, had they an interest in doing so. The great advantage to be had in being occasionally a member of different sects, is that acquaintances are made in each, and retained by a little tact and hospitality, no matter into whatever religious denomination you may afterwards pass. Indeed, the success of one of our first surgeons is owing to a calculation and tact of this sort; others have identified themselves at once with the high church and corporation religion of the town, and have generally been the most successful. They embarked at once with the strong party in the days of profligate corporation expenditure and rule, and have kept steady and true to their party and interest."

It may be as Dr. Collins says—his contemporaries may be calculating, pale-faced hypocrites, Cantwells who preach and purloin at the same instant —this may be true, most strictly true —but the question will arise, is Dr. Collins justified in publicly announcing it? The honourable reader may inquire what right Dr. Collins can claim to the office of a censor—why *he* should step forward to denounce his brethren as rogues and vagabonds. Yes, as rogues and vagabonds—perjurers who might experience and would merit the vengeance of the law. That the accusation is so gross is evident from a passage we proceed to quote.

"Others, again, stick their advertisements of lecturing in the medical library, the shops, the newspapers, and other places of notoriety, without giving or intending a lecture. In fact, this custom is getting very much into vogue: I wish it were more general, as, however quackish it may be, it can never injure the profession like the free-school system. The lecturers, when reproached with admitting persons to their lectures who never pay, though able to pay, say they do no harm, as they never give certificates of attendance without being paid. This may be so, for aught I know; but there was one gentleman, now deceased, in the habit of giving certificates of attendance on lectures he never delivered: he differed from others, inasmuch as he got paid for lectures he never gave. Even in his last sickness, a few hours before his death, for the sake of the money, and to oblige a friend, he put his name to one of those fictitious certificates; and yet he was a *respectable* man, a graduate of Cambridge, and a man of very considerable property."

If Dr. Collins has already gone so far, he may possibly be asked to proceed a little farther—to name the criminal he has so darkly painted. We can scarcely believe that any man would venture to manufacture false certificates in open day, as the culprit to whom Dr. Collins has alluded, must of course have done.

It is evident that the letter is of such a nature, as to render a rejoinder on the part of some member of the schools of Liverpool absolutely necessary. That it will have received an answer, of some kind, before the present notice is published, we can entertain but little doubt; and we should not be surprised, if in the heat of warfare, Dr. Collins experiences some ugly wounds. His antagonists may possibly employ the poisoned weapon he has used himself, and attack his motives, his character, and reputation. Strong in the consciousness of ill-natured truth, Dr. Collins may perhaps repel the assault.

Our business is not so much with Dr. Collins and his brethren, as with the question of permitting medical education to be exclusively provincial.—There is much to be said upon both sides, but, looking at the subject as dispassionately as possible, we are decidedly averse to such permission being granted. Whatever may be the talents, whatever the assiduity of provincial surgeons and physicians, their metropolitan brethren must always possess more extensive opportunities of becoming thoroughly acquainted with a science, which, in its practical application, is essentially founded on experience. Perhaps this is more strictly true of surgery than physic. The London surgeon in extensive practice *must* see and do much more than his provincial brother. The numbers of patients who repair from the country to London for medical advice, are the means of affording valuable information to those whom they consult.

Independent of the value of London tuition, the variety is advantageous. Were successive generations of provincial practitioners to be born, bred, educated, and established in the same county or town, we think there can be little question that the race would not thrive. The breed must be crossed in the moral world as in the animal, in order to prevent degeneration. Is there a provincial surgeon, possessed of means and ambitious of distinction, who would not voluntarily assist or complete his professional education in London? We venture to say that all would who could, and what common sense and self-interest would prompt for the benefit of one, must be equally adapted for the advantage of the many.

But the most important argument remains behind. It may be granted that no real scientific improvement results from attendance on the lecturers in London—that the country teachers are adapted for their office, yet still it would admit of more than doubt, if increased facilities and diminished expence to the student of medicine are really calculated to benefit the profession.

In the present constitution of society, what the world calls respectability is essential to the interests of classes as of individuals. That respectability may possibly be measured by a vicious standard—and general education, some degree of property, and gentlemanly manners, may not be the best tests of professional capacity. But, till the world is altered for the better, we must take it as it is; and, without interfering with the Owenites or St. Simonians, or any other order of philosophic philanthropists, we must, we are afraid, accommodate ourselves to circumstances. Now, the public complain that we are not on a par, in point of respectability, with the kindred professions of divinity and law. Those professions have more volunteers from the upper classes, they take the best men from the schools and the universities. If we render our education cheaper, our degrees more accessible than they are at present, we offer a bounty for the services of low-born, ill-bred recruits, who will swamp us by their numbers, and degrade us by their characters.

The object of the legislator and the friend to the profession should be to enhance its real respectability. This can only be done by thinning its numbers, and augmenting the acquirements of its members. It will not be effected by sending cryers to the lanes and highways, and bidding every beggar to the feast. If our College examinations were extremely rigid, instead of being, as they are, extremely lax—if the age of the candidate were required to be greater than it is at present—if some efficient guarantee were obtained for a *good* preliminary education—then we might be tempted to assent to the doctrine that it matters nothing where the actual knowledge is procured. But the present checks on the admission of unworthy persons are so slight, that we cannot afford to spare one.

---

### Baronetcy of Mr. Brodie.

The newspapers and the weekly medical journals will probably have informed the great majority of our readers, that his Majesty has been pleased to confer on Mr. Brodie the honor of the Baronetcy. Sir Benjamin Collins Brodie, for that is the present title of this eminent surgeon, has honorably merited this mark of the favor and discernment of his sovereign. The laurel-wreath was already on the brow; it has been merely gilt.

